# Social isolation and risk of heart disease and stroke: analysis of two large UK prospective studies

**DOI:** 10.1016/S2468-2667(20)30291-7

**Published:** 2021-03-02

**Authors:** Robert W Smith, Isobel Barnes, Jane Green, Gillian K Reeves, Valerie Beral, Sarah Floud

**Affiliations:** a Cancer Epidemiology Unit, Nuffield Department of Population Health, University of Oxford, Oxford, UK; b Upstream Lab, St Michael's Hospital, Toronto, ON, Canada; c Department of Family and Community Medicine, University of Toronto, Toronto, ON, Canada

## Abstract

**Background:**

Social isolation has been associated with increased risk of coronary heart disease and stroke. However, it is unclear whether the associations differ between fatal and non-fatal events or by the type of isolation (living alone or having few social contacts). We aimed to examine these associations in two large UK prospective cohorts.

**Methods:**

Million Women Study and UK Biobank participants without previous coronary heart disease or stroke who provided data in median year 2010 (IQR 2009–2011) on social contacts were included in this prospective analysis. Participants were followed up to median year 2017 (2017–2017) by electronic linkage to national hospital and death records. Risk ratios (RRs) were calculated using Cox regression for first coronary heart disease and stroke event (overall, and separately for hospital admission as the first event and for death without an associated hospital admission as the first event) by three levels of social isolation (based on living alone, contact with family or friends, and group participation) adjusted for age, sex, study, region, deprivation, smoking, alcohol intake, body-mass index, physical activity, and self-rated health.

**Findings:**

938 558 participants were included in our analyses (mean age 63 years [SD 9]): 481 946 participants from the Million Women Study (mean age 68 years [5]) and 456 612 participants (mean age 57 years [8]) from UK Biobank. During a mean follow-up period of 7 years (2), 42 402 first coronary heart disease events (of which 1834 were fatal without an associated hospital admission) and 19 999 first stroke events (of which 529 were fatal without an associated hospital admission) occurred. Little, if any, association was found between social isolation and hospital admission for a first coronary heart disease or stroke event (combined RR for both studies 1·01 [95% CI 0·98–1·04] for coronary heart disease and 1·13 [1·08–1·18] for stroke, when comparing the most isolated group with the least isolated group). However, the risk of death without an associated hospital admission was substantially higher in the most isolated group than the least isolated group for coronary heart disease (1·86 [1·63–2·12]) and stroke (1·91 [1·48–2·46]). For coronary heart disease or stroke death as the first event, RRs were substantially higher (test for heterogeneity, p=0·002) for participants living alone versus those not living alone (1·60 [1·46–1·75]) than for those with fewer versus more contact with family, friends, or groups (1·27 [1·16–1·38]). These findings did not differ greatly between studies, or by self-rated health.

**Interpretation:**

Social isolation seems to have little direct effect on the risk of developing a first coronary heart disease or stroke. By contrast, social isolation substantially increases the risk that the first such event is fatal before reaching hospital, particularly among people who live alone, perhaps because of the absence of immediate help in responding to an acute heart attack or stroke.

**Funding:**

UK Medical Research Council, Cancer Research UK.

## Introduction

Social isolation has emerged as a public health priority.^1^, ^2^ Although no established definition exists, social isolation is generally considered as having infrequent social contact with family, friends or groups, or living alone. The results of a 2016 meta-analysis aimed at assessing the association between social isolation and incident coronary heart disease and stroke^3^ are difficult to interpret because the analysis included studies that combined indices of both social isolation and loneliness. Since 2016, two relevant prospective studies have been published: one study found no association between social isolation and either coronary heart disease or stroke incidence after adjustment for standard vascular risk factors,^4^ and the other study found social isolation was associated with fatal coronary heart disease, but not with non-fatal myocardial infarction.^5^ Other evidence on the association between social isolation and death from coronary heart disease and stroke is sparse, since other studies combined deaths from all circulatory conditions and were often small in size.^6^, ^7^, ^8^ Therefore, we aimed to assess whether social isolation alone affects the risk of developing coronary heart disease or stroke, whether associations differ between fatal and non-fatal disease, and whether associations differ by type of social isolation (ie, living alone, contact with family or friends, or participating in group activities) in two large UK prospective studies: the Million Women Study and UK Biobank.

**Research in context**

**Evidence before this study**

A 2016 meta-analysis of studies that combined indices of social isolation and loneliness reported significant associations between these factors and incident coronary heart disease and incident stroke; however, the analysis did not assess the effect of social isolation alone. We searched MEDLINE from Jan 1, 1979, to Jan 6, 2020, without language restrictions, for prospective studies that assessed the association between social isolation (measured using indices of social contact frequency, group participation, and living alone or marital status) and incidence of coronary heart disease and stroke in adult populations. We used the terms “heart” OR “coronary” OR “artery” OR “ischem*” OR “ischaem*” OR “myocard*” adjacent to “disease” OR “attack” OR “event” OR “infarct*”, OR myocardial ischaemia OR cerebrovasc* OR stroke, AND “social isolation” OR “social support” OR “social integration”. Our search yielded fewer than ten studies. We identified two relevant prospective studies published since 2016: one study reported no association between social isolation and coronary heart disease and stroke after adjustment for standard vascular risk factors and the other study found social isolation was associated with fatal coronary heart disease, but not with non-fatal myocardial infarction.

**Added value of this study**

Our analyses included almost 1 million participants from two large UK prospective studies, the Million Women Study and UK Biobank. We used an index to define three levels of social isolation (based on living alone, contact with family or friends, and group participation). In the two studies combined, during a mean of 7 years (SD 2) of follow-up, 42 402 first coronary heart disease events and 19 999 first stroke events were observed, of which 1834 coronary heart disease events and 529 stroke events were fatal without an associated hospital admission. We found no association between social isolation and risk of a first coronary heart disease or first stroke that resulted in a hospital admission. By contrast, the risk of death among individuals without an associated hospital admission for coronary heart disease or stroke was substantially higher in the most isolated group than the least isolated group. These elevated risks for fatal events were considerably greater for individuals living alone than for those with little versus more social contact with family, friends, or groups. Results were similar when participants from each study were analysed separately.

**Implications of all the available evidence**

Results from this analysis of two large prospective studies and from other prospective studies indicate that social isolation has little direct effect on the risk of developing coronary heart disease or stroke. By contrast, social isolation contributes to the likelihood of dying from these conditions before reaching hospital, particularly among individuals who live alone, perhaps due to the absence of immediate help in responding to an acute coronary heart disease event or an acute stroke. A randomised trial should be considered to assess the effect of personal emergency alarms on mortality for people who live alone and are at risk of coronary heart disease or stroke.

## Methods

### Study design and participants

For this analysis, we obtained data from two UK prospective observational studies, the Million Women Study^9^ and UK Biobank.^10^ The design, methods, questionnaires, ethical approvals, and data access policies for the Million Women Study and UK Biobank are available online. For each study, participants provided written consent for participation in the study and follow-up through their medical records.

In median year 1998 (IQR 1997–1999), 1·3 million women invited for National Health Service (NHS) breast cancer screening at 66 screening centres in England and Scotland, were recruited to the Million Women Study. Participants completed a postal questionnaire at recruitment and subsequent questionnaires were sent every 3–5 years. A questionnaire completed in median year 2011 (IQR 2010–2012), included questions about social contact for the first time, and was the baseline for these analyses. In median year 2009 (IQR 2008–2009), 502 656 participants were recruited into UK Biobank and were asked to complete a touchscreen questionnaire, which included questions on social contact, and was the baseline for these analyses. The median baseline for both studies combined was 2010 (IQR 2009–2011).

### Procedures

The social isolation index used in the Million Women Study was constructed from four questions: question 1, how many people live in your household? (number of people including you; 1 point for living alone); question 2, how often do you contact (eg phone, meet, email) family; question 3, how often do you contact (eg phone, meet, email) friends; question 4, how often do you contact (eg phone, meet, email) groups (eg, religious groups, Women's Institute, fitness, adult education). The possible responses for questions 2–4 were: rarely or never, monthly, weekly or fortnightly, or most days (1 point was given for answering rarely or never, or monthly in response to both question 2 and question 3, and 1 point was given for rarely or never, or monthly in response to question 4). The social isolation index used in the UK Biobank was constructed from three questions: question 1, including yourself, how many people are living together in your household (1 point was given for living alone); question 2, how often do you visit friends or family or have them visit you (1 point was given for answering about once a month, once every few months, never or almost never, or no friends or family outside household); question 3, which of the following (sports club or gym, pub or social club, religious group, adult education class, other group activity) do you engage in once a week or more often (1 point was given for answering none of the above). Individual scores were summed to calculate an overall score ranging from 0 to 3. For the purposes of this analysis, individuals from both studies were defined as least isolated if they scored 0, moderately isolated if they scored 1, and most isolated if they scored 2 or 3 (scores of 2 or 3 were grouped since few individuals had scores of 3). This index is similar to other indices of social isolation, including the Berkman-Syme social network index.^7^, ^11^, ^12^

Participants in both studies were registered with the NHS. Each individual's unique NHS number (or equivalent) and date of birth were used to link to routinely collected NHS data on hospital admissions, deaths, and emigrations. Electronic linkage was done by NHS Digital in England, NHS Central Register Scotland, and Information Services Division Scotland, and Secure Anonymised Information Linkage Wales. Hospital diagnoses for each admission were coded using the International Classification of Diseases, 10th revision (ICD-10).^13^ The main outcomes were first coronary heart disease event (ICD-10 codes I20–I25) and first stroke event (I60–I69). We subdivided first events into hospital admission as the first event or death with no associated hospital admission as the first event. First events for participants who died on the first day of their hospital admission were classified as death as the first event. We calculated person-years from the date participants answered the social contact questions to date of first event, death, cessation of NHS registration, or cessation of follow-up, whichever occurred first. In the Million Women Study, participants were followed up until Jan 1, 2018. In UK Biobank, participants in Wales were followed up until March 1, 2016, participants in Scotland were followed up until Nov 1, 2016, and participants in England were followed up until Jan 1, 2018. The median end of follow-up for both studies combined was 2017 (IQR 2017–2017).

### Statistical analysis

We restricted analyses to participants who answered all questions on social contact and did not have previous self-reported or recorded hospital admission for coronary heart disease or stroke. Cox regression analyses were used to calculate hazard ratios (equivalent to, and referred to hereafter, as risk ratios [RRs]) and 95% CIs to compare participants classified according to various prespecified indices of social isolation. Where appropriate, results for both studies and for both outcomes of interest were combined. Additionally, we used the constituent measures of the index (eg, living alone, contact with family or friends, group participation) to compare individuals who lived alone with those who did not, and to compare those with infrequent contact with family, friends or groups versus those with more frequent contact (scores of 1–2 *vs* 0 on the questions about family or friends and about groups). In the regression models, attained age was the underlying time variable and analyses were stratified by study (in combined analyses) and sex (in UK Biobank and combined analyses) and adjusted for region of recruitment (appendix p 3) and area deprivation quintiles (based on the Townsend Index^14^). We further adjusted for the following variables at baseline: cigarette smoking (never, past, current <15 cigarettes per day, current ≥15 cigarettes per day); alcohol intake (0, 1–7 units per week, ≥7 units per week); physical activity (strenuous or moderate physical activity; <1 day per week, 1–3 days per week, ≥4 days per week), body-mass index (BMI; <25, 25–<30, ≥30 kg/m^2^); and self-rated health (excellent, good, fair, poor). For each adjustment variable, missing values were assigned a separate category. Categorical adjustment variables were used throughout to minimise any undue influence of extreme values. χ^2^ tests were used to assess heterogeneity. To quantify the extent to which an association could be accounted for by each adjustment factor, the reduction in the likelihood-ratio χ^2^ test statistic was calculated.^15^ We also did a sensitivity analysis of the Million Women Study data to minimise effects of changes in health behaviours caused by social isolation, using information on these factors recorded in median year 1998, 13 years before the questions on social isolation were included in the study questionnaire; no such data were available for UK Biobank. In other sensitivity analyses, we made additional adjustments for possible factors that could lie on the causal pathway from social isolation to coronary heart disease or stroke: history of high blood pressure, history of diabetes, use of cholesterol-lowering medication, and a history of depression (appendix p 8). Analyses were also done separately in those with poor or fair and good or excellent self-rated health. Analyses of sex differences were restricted to participants included in the UK Biobank. We did another sensitivity analysis to assess the effect of excluding from the deaths without an associated hospital admission any deaths of participants who died on the first day of their hospital admission. All analyses used Stata (version 15.1).

### Role of the funding source

The funders of the study had no role in the study design, data collection, data analysis, data interpretation, or writing of the report.

## Results

Our analyses included 938 558 participants (mean age 63 years [SD 9]): 481 946 participants from the Million Women Study (mean age 68 years [5]) and 456 612 participants (mean age 57 years [8]) from UK Biobank, of whom 255 293 (56%) were women. Of the 938 558 participants included, 129 986 (14%) were classified as most isolated, 429 541 (46%) as moderately isolated, and 379 031 (40%) as least isolated at baseline. Compared with the least isolated group, participants in the most isolated group were more likely to reside in a deprived area and to be obese, current smokers, physically inactive, and in poor health (table). A higher proportion of participants reported living alone in the Million Women Study (118 723 [25%] of 481 946 participants) than in the UK Biobank (83 649 [18%] of 456 612 participants). Although the prevalence of some baseline characteristics differed between the two studies, the distribution of the characteristics according to the level of social isolation followed a similar pattern in each study (appendix p 4).

**Table:** Baseline characteristics of participants included in the analyses (n=938 558)*, by level of social isolation and first coronary heart disease and stroke event

		**Least isolated (n=379 031)**	**Moderately isolated (n=429 541)**	**Most isolated (n=129 986)**
**Baseline characteristics**				
Age at baseline, years		62·0 (8·6)	63·3 (8·7)	62·8 (9·4)
Sex				
	Men	88 582 (23·4%)	80 734 (18·8%)	32 004 (24·6%)
	Women	290 449 (76·6%)	348 807 (81·2%)	97 982 (75·4%)
Most deprived quintile†		45 108 (12·0%)	73 944 (17·3%)	33 232 (25·7%)
Current smoker		19 531 (5·2%)	35 714 (8·4%)	17 583 (13·8%)
Obesity (BMI ≥30 kg/m^2^)		73 819 (19·7%)	97 506 (23·2%)	32 191 (25·4%)
≥7 units of alcohol per week		135 279 (38·7%)	123 681 (32·3%)	32 923 (29·2%)
Rarely or never exercise		28 446 (7·9%)	51 600 (13·3%)	21 762 (18·8%)
Poor or fair self-rated health		57 948 (15·4%)	92 539 (21·7%)	38 143 (29·6%)
**Follow-up for first coronary heart disease event**				
Follow-up duration, years per participant		7·2 (1·5)	7·0 (1·6)	7·0 (1·7)
Person-years, 1000s		2737	3000	908
Number of first events		15 190	19 919	7293
	Number of hospital admissions as first event	14 714	19 067	6787
	Number of deaths without an associated hospital admission as first event	476	852	506
**Follow-up for first stroke event**				
Follow-up duration, years per participant		7·3 (1·4)	7·1 (1·5)	7·1 (1·6)
Person-years, 1000s		2769	3036	920
Number of first events		6513	9748	3738
	Number of hospital admissions as first event	6380	9490	3600
	Number of deaths without an associated hospital admission as first event	133	258	138

Data are mean (SD), n, or n (%). BMI=body-mass index.

* Data for Million Women Study and UK Biobank participants were combined; baseline characteristics of participants included in each study separately are shown in the appendix (p 4).

† According to the Townsend Index.^14^

**Figure 1:**
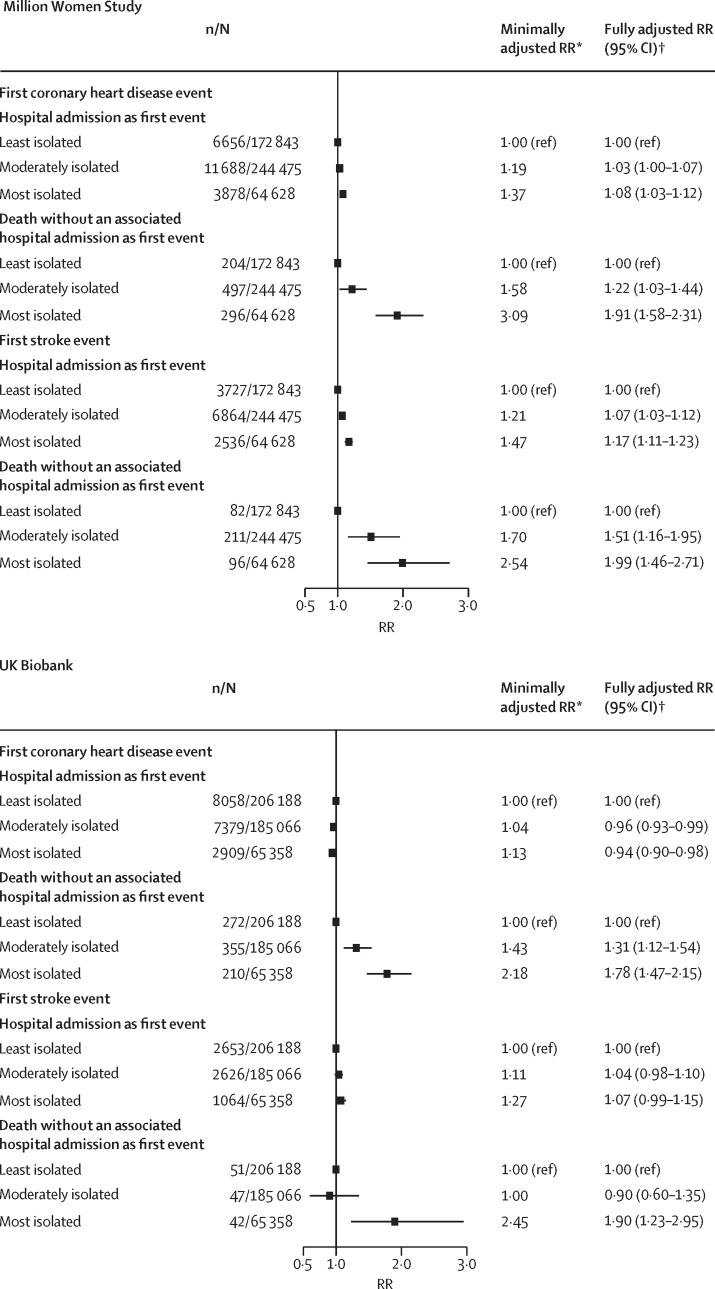
Study-specific associations between first coronary heart disease and first stroke events and level of social isolation, by first event type (hospital admission or death without an associated hospital admission) RR=risk ratio. *Adjusted for age, sex (in UK Biobank), region, and deprivation. †Adjusted for age, sex (in UK Biobank), region, deprivation, smoking, alcohol intake, body-mass index, physical activity, and self-rated health.

In the two studies combined, during a mean follow-up period of 7 years (SD 2), 42 402 first coronary heart disease events and 19 999 first stroke events occurred. In the Million Women Study, 23 219 first coronary heart disease events and 13 516 first stroke events occurred during a mean of 6 years (SD 2) of follow-up, and in UK Biobank, 19 183 first coronary heart disease events and 6483 first stroke events occurred during a mean duration of 8 years (1) of follow-up. Overall weak, if any, study-specific associations were found between social isolation and first coronary heart disease event or first stroke event (appendix p 5). However, when results were subdivided by type of first event (hospital admission or death with no associated hospital admission), social isolation was strongly associated with death as the first event (figure 1). For deaths from coronary heart disease as the first event, the risk for the most isolated group was higher than for the least isolated group in both studies (RR 1·91 [95% CI 1·58–2·31] in the Million Women Study; 1·78 [1·47–2·15] in UK Biobank); and similarly, risks in the most isolated group were higher in both studies for deaths from stroke as the first event (1·99 [1·46–2·71] in the Million Women Study; 1·90 [1·23–2·95] in UK Biobank; figure 1). By contrast, any association between social isolation and hospital admission as first event was weak (figure 1).

**Figure 2:**
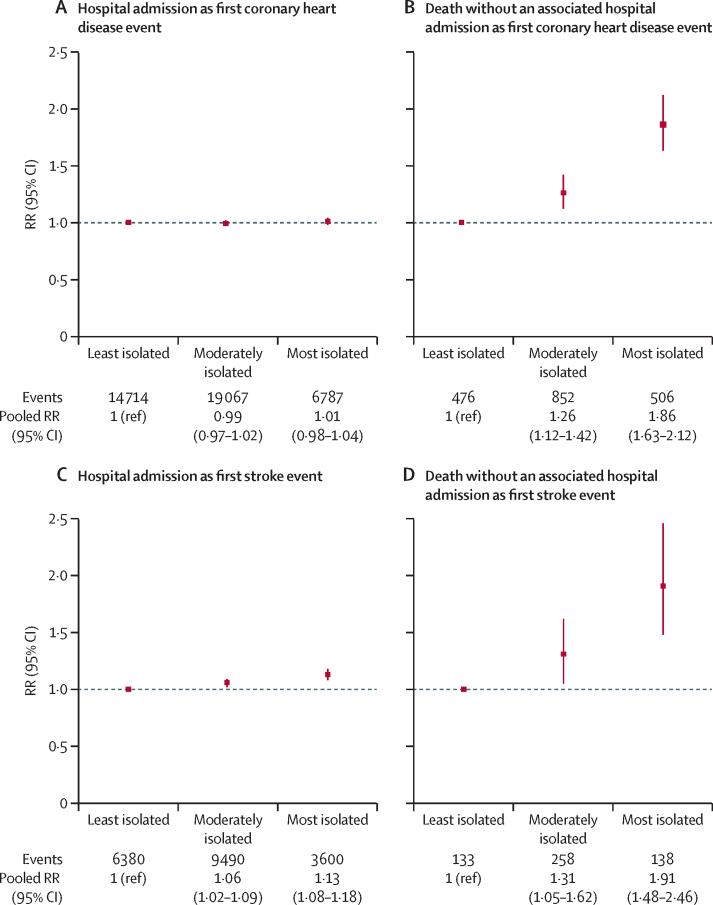
Associations between first coronary heart disease and first stroke event and level of social isolation, by first event type (hospital admission or death without an associated hospital admission; both studies combined) RRs adjusted for age, sex, study, region, deprivation, smoking, alcohol intake, body-mass index, physical activity, and self-rated health. RR=risk ratio.

Since findings were similar in both studies, we combined their results for some analyses (figure 2). For first events resulting in a hospital admission when comparing the most isolated group with the least isolated group, little association was identified between level of social isolation and risk of coronary heart disease (pooled RR 1·01 [0·98–1·04]) or stroke (1·13 [1·08–1·18]; figure 2), whereas the risks were substantially greater for death as the first event from coronary heart disease (1·86 [1·63–2·12]) or from stroke (1·91 [1·48–2·46]; test for heterogeneity between fatal and non-fatal disease, p<0·0001 for coronary heart disease and p=0·0003 for stroke).

In sensitivity analyses, the results for death as a first event were similar to those in the main analyses after excluding the deaths of the participants who died on the day of hospital admission (84 for coronary heart disease; 123 for stroke): the risk of fatal events remained higher in the most isolated group than in the least isolated group for coronary heart disease (pooled RR 1·85 [1·61–2·11]) and stroke (2·39 [1·79–3·20]).

Further sensitivity analyses indicated the robustness of results for death as the first coronary heart disease or stroke event (appendix pp 6–8). Residual confounding by smoking, BMI, alcohol intake, physical activity, and self-rated health did not seem to account for the association (appendix p 6). The possibility that social isolation had altered these lifestyle and personal factors and hence that they might be mediators of the association between social isolation and coronary heart disease or stroke was not supported by analyses which used measures of these factors recorded more than a decade before social isolation was recorded and showed similar results to the main analyses (appendix p 7). Adjustment for potential mediators of the association between social isolation and coronary heart disease or stroke, such as high blood pressure, diabetes, high cholesterol, and depression, did not materially change the RRs (appendix p 8).

**Figure 3:**
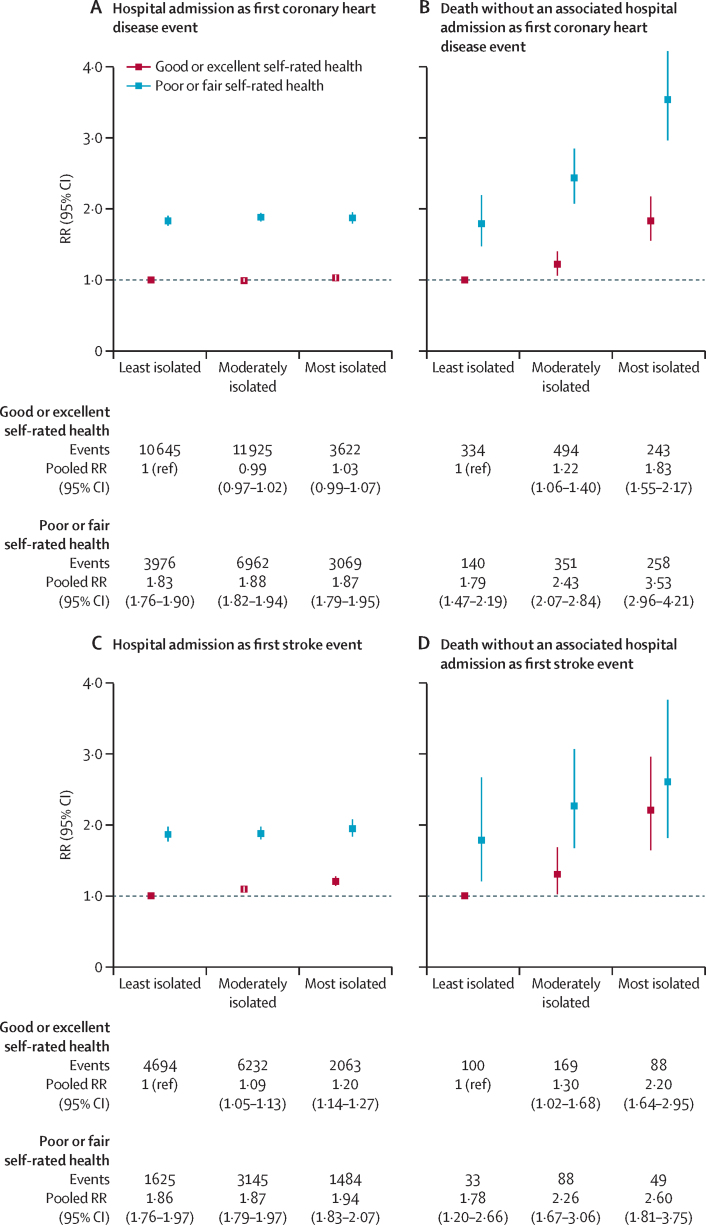
Associations between first coronary heart disease and first stroke event and level of social isolation and self-rated health, by first event type (hospital admission or death without an associated hospital admission; both studies combined) RRs adjusted for age, sex, study, region, deprivation, smoking, alcohol intake, body-mass index, and physical activity. RR=risk ratio.

Social isolation was more common in those who rated their health as poor or fair than in those who rated their health as good or excellent (table), and those who rated their health as poor or fair were at greater risk of coronary heart disease or stroke (figure 3). Despite the higher incidence of disease among participants with poor or fair health, the associations between social isolation level and each coronary heart disease and stroke outcome were broadly similar with regard to the direction of the effect among participants who rated their health as poor or fair and those who rated their health as good or excellent (figure 3).

**Figure 4:**
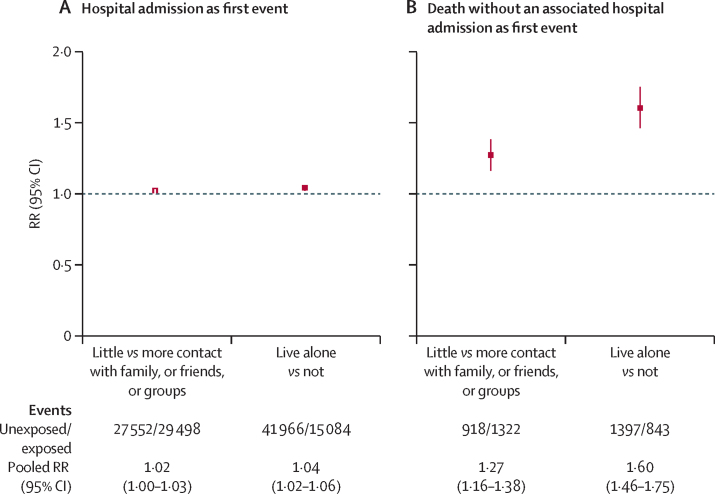
Associations between first coronary heart disease or first stroke event and living alone and amount of contact with family, friends, or groups, by first event type (hospital admission or death without an associated hospital admission; both studies combined) RRs adjusted for age, sex, study, region, deprivation, smoking, alcohol intake, body-mass index, physical activity and self−rated health. RR=risk ratio.

When analysing the measures that contributed to social isolation and combining results for coronary heart disease or stroke as the first event (because RRs were similar for both conditions), there remained little evidence of an association between social isolation and risk of hospital admission as the first event (figure 4). By contrast, for death as the first event, the risk was significantly greater for individuals who lived alone versus those who did not (RR 1·60 [95% CI 1·46–1·75]) compared with individuals who had little contact with family, friends, or groups versus those who had more contact with family, friends, or groups (1·27 [1·16–1·38]; test for heterogeneity, p=0·002; figure 4). No material differences between the two studies were identified with regard to these associations with the measures that contributed to the index of social isolation (appendix p 9). Little difference was identified in the associations by sex (appendix p 10).

## Discussion

This analysis of two large UK prospective studies showed that social isolation was associated with a substantial increase in the risk of first coronary heart disease events and first stroke events that resulted in death without an associated hospital admission. By contrast, little or no association was identified between social isolation and the risk of first coronary heart disease or stroke events that resulted in a hospital admission. Considering the different measures that contributed to the index of social isolation used, living alone was more strongly associated with the risk of a first fatal coronary heart disease event or a first fatal stroke than was having little contact with friends, family, or groups. Taken together, these findings suggest that social isolation has little direct effect on the risk of developing coronary heart disease or stroke, but that social isolation increases the likelihood that a first event is fatal before reaching hospital, perhaps because of the absence of immediate help in response to an acute event such as a heart attack or stroke.

One strength of this analysis is that when analysed separately, the findings from the Million Women Study and UK Biobank were similar, even though their characteristics were different and the questionnaires used to assess social isolation were slightly different. Participants in the Million Women Study were asked about any contact with others (by email, phone, and in person), whereas UK Biobank participants were asked only about visiting or having a visit from family or friends in person. Participants included in the Million Women Study were all women and were about a decade older than UK Biobank participants and more likely to live alone. Other strengths include the prospective design of both studies, which reduces the risk of recall bias, and the adjustment for the most relevant confounders. A further strength is the large number of events that occurred during follow-up in both studies, which provided adequate power to compare the risks of fatal and non-fatal coronary heart disease and stroke events associated not only with different levels of social isolation, but also with different components of the social isolation index. A limitation of our analyses was that around 10% of UK Biobank participants might have also been included in the Million Women Study; however, although it was not possible to identify the overlapping individuals, this relatively small duplication would likely have had little effect on the main findings.

The main findings of our analysis were consistent across the two studies, and are also consistent with the findings from other prospective studies that examined associations of social isolation by itself with coronary heart disease and stroke,^5^, ^16^, ^17^, ^18^, ^19^, ^20^ including a previous report of UK Biobank data with shorter follow-up.^4^ However, these results cannot be compared with findings from a 2016 meta-analysis, which included studies that combined measures of social isolation with measures of loneliness.^3^ To our knowledge, only two previously published studies have assessed social isolation by itself and separately for fatal and for non-fatal first coronary heart disease and first stroke events and results from both studies were consistent with our findings. The US Health Professionals Study of 28 369 men (142 fatal coronary heart disease events and 618 non-fatal myocardial infarction events), reported a statistically significant association between social isolation and fatal coronary heart disease, but not with non-fatal myocardial infarction.^17^ The US Nurses' Health Study of 76 362 women (408 fatal coronary heart disease events and 1964 non-fatal myocardial infarction events) also reported a statistically significant association between social isolation and fatal disease, but not non-fatal disease.^5^

It has been suggested that the associations between social isolation and fatal disease could be mediated by inflammation,^5^ or that they could be due to the absence of another person who would be able to assist with seeking health care during acute coronary heart disease events.^21^ The view that the association is due to the absence of another person is supported by our finding that the magnitude of risk of fatal events was greater for individuals living alone than for those who had little social contact with others. Consideration should be given to a randomised trial to investigate the effect of some form of personal emergency alarm on mortality for people who live alone and are at risk of coronary heart disease or stroke. Although studies of personal alarms in the community have been reported, they tend to have small samples and often focus on the risk of falls and on user experience rather than on cardiovascular mortality or hospital admissions.^22^

The substantial difference between the risk for fatal and non-fatal disease as a first event suggests that these differences are unlikely to be due to confounding by risk factors for coronary heart disease or stroke.^4^, ^5^, ^7^ Although social isolation was more common in individuals who rated their health as poor or fair—who themselves were at a considerably increased risk of coronary heart disease, stroke, and all-cause mortaity^23^—associations between social isolation and coronary heart disease and stroke were similar when individuals with poor or fair and excellent or good self-rated health were assessed separately. It has been suggested that social isolation might increase the likelihood of smoking and hence that smoking could mediate the association between social isolation and coronary heart disease and stroke,^5^ but there are several reasons why this seems unlikely: the findings differ for fatal and non-fatal disease, few smokers start smoking after early adulthood when social isolation would be most relevant,^24^ and there was little change in the findings when we used data on smoking habits reported about a decade before social isolation was assessed. Some studies have reported that associations with social isolation might be greater in men than women^4^, ^7^, ^8^ and others have reported the opposite;^6^ however, in our analyses, we found little material difference in the association with fatal disease by sex.

Overall, our results and those from other prospective studies suggest that social isolation itself has little or no direct effect on the risk of developing coronary heart disease or stroke. Such findings are contrary to a common public health message that social isolation increases the risk of developing vascular disease.^1^, ^25^, ^26^, ^27^ However, we found that social isolation, in particular living alone, was associated with a higher risk of first coronary heart disease or stroke events being fatal before reaching hospital, perhaps because affected individuals have no one immediately available who can help in responding to these acute illnesses.

## Data sharing

Million Women Study and UK Biobank data are available online.
