# Supplementary material for: Social isolation and risk of heart disease and stroke: analysis of two large UK prospective studies
Source: Lancet Public Health. 2021 Mar 2;6(4):e232–9. doi: 10.1016/S2468-2667(20)30291-7 (PMC7994247; doi:10.1016/S2468-2667(20)30291-7)
Supplement: Supplementary appendix [file mmc1.pdf]

THE LANCET
Public Health

Supplementary appendix

This appendix formed part of the original submission and has been peer reviewed. We post it as supplied by the authors.

Supplement to: Smith RW, Barnes I, Green J, Reeves GK, Beral V, Floud S. Social isolation and risk of heart disease and stroke: analysis of two large UK prospective studies. *Lancet Public Health* 2021; published online March 1. http://dx.doi.org/10.1016/S2468-2667(20)30291-7.

This blank page is inserted to rectify the appendix page numbering during the Lancet Public Health editorial process

CONTENTS -

Page	
3	Information on adjustment for region in the Million Women Study and UK Biobank
4	eTable 1. Study-specific baseline characteristics by social isolation and details of follow-up for incident coronary heart disease and stroke
5	eFigure 1. Study-specific Risk Ratios and 95% CIs for any first coronary heart disease events and any first stroke events in relation to three levels of social isolation
6	eTable 2. Risk Ratios and 95% CIs for first coronary heart disease and stroke events in relation to three levels of social isolation in both studies combined and subdivided by whether the first event was a hospital admission or a death without an associated hospital admission: effect of adjustments
7	eTable 3. Risk Ratios and 95% CIs for first coronary heart disease and stroke events in relation to three levels of social isolation, subdivided by whether the first event was a hospital admission or a death without an associated hospital admission, with adjustments taken from recruitment in 1998, in Million Women Study only
8	eTable 4. Risk Ratios and 95% CIs for first coronary heart disease and stroke events in relation to three levels of social isolation in both studies combined, subdivided by whether the first event was a hospital admission or a death without an associated hospital admission: additional adjustments
9	eFigure 2. Study-specific Risk Ratios and 95% CIs for coronary heart disease and stroke first events in relation to living alone and to having little contact with family/friends/groups, subdivided by whether the first event was a hospital admission or a death without an associated hospital admission
10	eTable 5. Risk Ratios and 95% CIs for first coronary heart disease and stroke events in relation to three levels of social isolation, subdivided by sex and by whether the first event was a hospital admission or a death without an associated hospital admission, in UK Biobank only
11	Membership of the Million Women Study Advisory Committee, the Million Women Study coordinating centre staff, NHS Breast Cancer Screening Programme collaborating centres

Information on adjustment for region in the Million Women Study and UK Biobank

The regions in both the Million Women Study and UK Biobank were classified according to the Cancer Registry regions in England: Oxford, Thames, West Midlands, North Yorkshire, Trent, North West (Mersey), North West (Manchester). Other regions were Scotland in both the Million Women Study and UK Biobank; and Wales in UK Biobank.

eTable 1. Study-specific baseline characteristics by social isolation and details of follow-up for incident coronary heart disease and stroke

	Million Women Study						UK Biobank					
	Least Isolated (n = 172,843)		Moderately Isolated (n = 244,475)		Most isolated (n = 64,628)		Least Isolated (n = 206,188)		Moderately Isolated (n = 185,066)		Most isolated (n = 65,358)	
**Baseline characteristics**												
Mean age at baseline (SD), years	68.0	(4.3)	68.5	(4.6)	69.5	(4.9)	57.0	(8.1)	56.5	(8.1)	56.2	(7.9)
Female, %	100.0		100.0		100.0		57.0		56.4		51.0	
Most deprived fifth, %	9.2		14.8		22.1		14.2		20.6		29.2	
Current smoker, %	2.5		6.9		12.4		7.5		10.5		15.1	
Obese, %	16.8		22.3		24.3		22.1		24.3		26.4	
7+ units per week of alcohol, %	27.7		25.1		21.9		47.5		41.3		35.8	
Rarely/never exercise, %	7.3		12.8		17.7		8.4		13.9		19.8	
Poor/fair self-rated health, %	11.2		19.4		26.8		18.9		24.7		32.4	
**Follow-up for first coronary heart disease event**												
Mean (SD) years of follow-up per participant	6.5	(1.4)	6.3	(1.5)	6.2	(1.6)	7.9	(1.3)	7.8	(1.3)	7.8	(1.4)
Total number of first events	6,860		12,185		4,174		8,330		7,734		3,119	
-Number of hospital admissions as first event	6,656		11,688		3,878		8,058		7,379		2,909	
-Number of deaths without an associated hospital admission as first event	204		497		296		272		355		210	
**Follow-up for first stroke event**												
Mean (SD) years of follow-up per participant	6.5	(1.3)	6.4	(1.4)	6.3	(1.5)	8.0	(1.1)	7.9	(1.2)	7.9	(1.2)
Total number of first events	3,809		7,075		2,632		2,704		2,673		1,106	
-Number of hospital admissions as first event	3,727		6,864		2,536		2,653		2,626		1,064	
-Number of deaths without an associated hospital admission as first event	82		211		96		51		47		42	

eFigure 1. Study-specific risk ratios (RRs) and 95% CIs for any first coronary heart disease events and any first stroke events in relation to three levels of social isolation

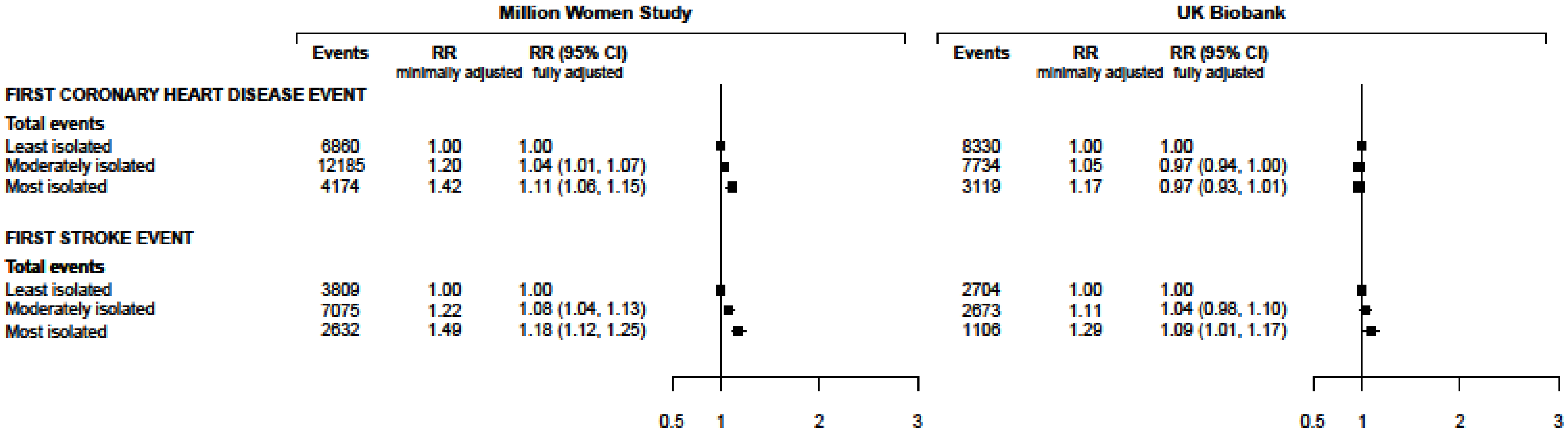

Minimally adjusted for age, sex (in UK Biobank), region, deprivation. Fully adjusted for age, sex (in UK Biobank), region, deprivation, smoking, alcohol intake, body mass index, physical activity and self-rated health

eTable 2. Risk ratios and 95% CIs for first coronary heart disease and stroke events in relation to three levels of social isolation in both studies combined and subdivided by whether the first event was a hospital admission or a death without an associated hospital admission: effect of adjustments

	Least isolated	Moderately isolated	Most isolated	LR	% Reduction in LR
**FIRST CORONARY HEART DISEASE EVENT**					
**Hospital admission as first event**					
Risk Ratio adjusted for:					
**Age, sex, region, deprivation only**	**1.00 (---)**	**1.11 (1.08-1.13)**	**1.24 (1.21-1.28)**	**220.0**	**---**
Age, sex, region, deprivation, smoking	1.00 (---)	1.08 (1.06-1.11)	1.18 (1.14-1.21)	123.8	44
Age, sex, region, deprivation, body mass index	1.00 (---)	1.09 (1.07-1.12)	1.22 (1.19-1.26)	181.9	17
Age, sex, region, deprivation, alcohol	1.00 (---)	1.09 (1.07-1.11)	1.20 (1.16-1.23)	148.4	33
Age, sex, region, deprivation, physical activity	1.00 (---)	1.08 (1.05-1.10)	1.18 (1.15-1.22)	124.9	43
Age, sex, region, deprivation, self-rated health	1.00 (---)	1.04 (1.01-1.06)	1.09 (1.06-1.12)	31.7	86
**All variables**	**1.00 (---)**	**0.99 (0.97-1.02)**	**1.01 (0.98-1.04)**	**1.1**	**100**
**Death without an associated hospital admission as first event**					
Risk Ratio adjusted for:					
**Age, sex, region, deprivation only**	**1.00 (---)**	**1.49 (1.33-1.67)**	**2.60 (2.29-2.96)**	**209.4**	**---**
Age, sex, region, deprivation, smoking	1.00 (---)	1.39 (1.24-1.56)	2.24 (1.96-2.55)	145.3	31
Age, sex, region, deprivation, body mass index	1.00 (---)	1.47 (1.31-1.64)	2.54 (2.24-2.89)	199.1	5
Age, sex, region, deprivation, alcohol	1.00 (---)	1.46 (1.30-1.63)	2.49 (2.19-2.84)	188.7	10
Age, sex, region, deprivation, physical activity	1.00 (---)	1.41 (1.26-1.59)	2.38 (2.09-2.71)	169.9	19
Age, sex, region, deprivation, self-rated health	1.00 (---)	1.38 (1.23-1.55)	2.24 (1.97-2.55)	147.4	30
**All variables**	**1.00 (---)**	**1.26 (1.12-1.42)**	**1.86 (1.63-2.12)**	**84.1**	**60**
**FIRST STROKE EVENT**					
**Hospital admission as first event**					
Risk Ratio adjusted for:					
**Age, sex, region, deprivation only**	**1.00 (---)**	**1.17 (1.14-1.21)**	**1.39 (1.34-1.45)**	**246.0**	**---**
Age, sex, region, deprivation, smoking	1.00 (---)	1.13 (1.10-1.17)	1.29 (1.23-1.34)	142.3	42
Age, sex, region, deprivation, body mass index	1.00 (---)	1.17 (1.13-1.20)	1.38 (1.32-1.44)	229.6	7
Age, sex, region, deprivation, alcohol	1.00 (---)	1.16 (1.12-1.20)	1.37 (1.31-1.42)	215.0	13
Age, sex, region, deprivation, physical activity	1.00 (---)	1.14 (1.10-1.17)	1.32 (1.27-1.38)	169.4	31
Age, sex, region, deprivation, self-rated health	1.00 (---)	1.11 (1.07-1.14)	1.24 (1.19-1.29)	102.1	58
**All variables**	**1.00 (---)**	**1.06 (1.02-1.09)**	**1.13 (1.08-1.18)**	**32.1**	**87**
**Death without an associated hospital admission as first event**					
Risk Ratio adjusted for:					
**Age, sex, region, deprivation only**	**1.00 (---)**	**1.48 (1.20-1.83)**	**2.48 (1.94-3.17)**	**52.0**	**---**
Age, sex, region, deprivation, smoking	1.00 (---)	1.40 (1.14-1.74)	2.21 (1.72-2.83)	38.6	26
Age, sex, region, deprivation, body mass index	1.00 (---)	1.48 (1.20-1.83)	2.46 (1.93-3.14)	50.9	2
Age, sex, region, deprivation, alcohol	1.00 (---)	1.46 (1.18-1.81)	2.43 (1.90-3.10)	49.1	6
Age, sex, region, deprivation, physical activity	1.00 (---)	1.41 (1.14-1.75)	2.29 (1.79-2.93)	42.9	17
Age, sex, region, deprivation, self-rated health	1.00 (---)	1.40 (1.13-1.74)	2.24 (1.75-2.86)	40.2	23
**All variables**	**1.00 (---)**	**1.31 (1.05-1.62)**	**1.91 (1.48-2.46)**	**25.1**	**52**

LR = likelihood ratio test statistic

eTable 3. Risk Ratios and 95% CIs for first coronary heart disease and stroke events in relation to three levels of social isolation, subdivided by whether the first event was a hospital admission or a death without an associated hospital admission, with adjustments taken from recruitment in 1998, in Million Women Study only

	Events	RR (95% CI) fully adjusted*	
**FIRST CORONARY HEART DISEASE EVENT**			
**Hospital admission as first event**			
Least isolated	6,656	1.00	---
Moderately isolated	11,688	1.04	(1.00,1.07)
Most isolated	3,878	1.08	(1.03,1.12)
**Death without an associated hospital admission as first event**			
Least isolated	204	1.00	---
Moderately isolated	497	1.26	(1.07,1.49)
Most isolated	296	2.04	(1.69,2.46)
**FIRST STROKE EVENT**			
**Hospital admission as first event**			
Least isolated	3,727	1.00	---
Moderately isolated	6,864	1.08	(1.04,1.12)
Most isolated	2,536	1.19	(1.13,1.25)
**Death without an associated hospital admission as first event**			
Least isolated	82	1.00	---
Moderately isolated	211	1.53	(1.18,1.99)
Most isolated	96	2.08	(1.53,2.83)

*Fully adjusted for age, region, deprivation, smoking, alcohol intake, body mass index, physical activity and self-rated health, with all adjustment variables measured at recruitment in 1998 except self-rated health.

eTable 4. Risk Ratios and 95% CIs for first coronary heart disease and stroke events in relation to three levels of social isolation in both studies combined, subdivided by whether the first event was a hospital admission or a death without an associated hospital admission: additional adjustments

	Least isolated	Moderately isolated	Most isolated
**FIRST CORONARY HEART DISEASE EVENT**			
**Hospital admission as first event**			
Risk Ratio adjusted for:			
**Fully adjusted**	**1.00 (---)**	**0.99 (0.97-1.02)**	**1.01 (0.98-1.04)**
Fully adjusted plus hypertension	1.00 (---)	0.99 (0.97-1.01)	1.00 (0.97-1.03)
Fully adjusted plus diabetes	1.00 (---)	0.99 (0.97-1.02)	1.01 (0.98-1.04)
Fully adjusted plus high cholesterol	1.00 (---)	0.99 (0.97-1.02)	1.01 (0.98-1.04)
Fully adjusted plus depression	1.00 (---)	0.99 (0.97-1.02)	1.01 (0.98-1.04)
**Adjusted for all variables**	**1.00 (---)**	**0.99 (0.97-1.01)**	**1.00 (0.97-1.03)**
**Death without an associated hospital admission as first event**			
Risk Ratio adjusted for:			
**Fully adjusted**	**1.00 (---)**	**1.26 (1.12-1.42)**	**1.86 (1.63-2.12)**
Fully adjusted plus hypertension	1.00 (---)	1.26 (1.12-1.41)	1.85 (1.62-2.11)
Fully adjusted plus diabetes	1.00 (---)	1.26 (1.12-1.41)	1.84 (1.61-2.10)
Fully adjusted plus high cholesterol	1.00 (---)	1.26 (1.12-1.41)	1.86 (1.63-2.12)
Fully adjusted plus depression	1.00 (---)	1.26 (1.12-1.42)	1.86 (1.62-2.12)
**Adjusted for all variables**	**1.00 (---)**	**1.25 (1.12-1.41)**	**1.83 (1.60-2.09)**
**FIRST STROKE EVENT**			
**Hospital admission as first event**			
Risk Ratio adjusted for:			
**Fully adjusted**	**1.00 (---)**	**1.06 (1.02-1.09)**	**1.13 (1.08-1.18)**
Fully adjusted plus hypertension	1.00 (---)	1.05 (1.02-1.09)	1.12 (1.08-1.17)
Fully adjusted plus diabetes	1.00 (---)	1.06 (1.02-1.09)	1.13 (1.08-1.18)
Fully adjusted plus high cholesterol	1.00 (---)	1.06 (1.02-1.09)	1.13 (1.08-1.18)
Fully adjusted plus depression	1.00 (---)	1.06 (1.02-1.09)	1.13 (1.08-1.18)
**Adjusted for all variables**	**1.00 (---)**	**1.05 (1.02-1.09)**	**1.12 (1.07-1.17)**
**Death without an associated hospital admission as first event**			
Risk Ratio adjusted for:			
**Fully adjusted**	**1.00 (---)**	**1.31 (1.05-1.62)**	**1.91 (1.48-2.46)**
Fully adjusted plus hypertension	1.00 (---)	1.30 (1.05-1.61)	1.89 (1.47-2.43)
Fully adjusted plus diabetes	1.00 (---)	1.30 (1.05-1.62)	1.90 (1.48-2.45)
Fully adjusted plus high cholesterol	1.00 (---)	1.30 (1.05-1.62)	1.91 (1.48-2.45)
Fully adjusted plus depression	1.00 (---)	1.31 (1.05-1.62)	1.90 (1.48-2.44)
**Adjusted for all variables**	**1.00 (---)**	**1.30 (1.05-1.61)**	**1.88 (1.46-2.42)**

Fully adjusted model = adjusted for age, sex, study, region, deprivation, smoking, alcohol intake, body mass index, physical activity and self-rated health. Four additional covariates (hypertension, diabetes, high cholesterol and depression) were then added separately and together to the fully adjusted model. Variables used for high blood pressure: self-reported history of high blood pressure (diagnosed by doctor or self-reported having been told they had a high blood pressure reading in last 5 years or self-reported ever having had high blood pressure) in Million Women Study; self-reported diagnosis by doctor in UK Biobank. Variables used for diabetes: self-reported diagnosis by doctor in the Million Women Study and UK Biobank. Variables used for high cholesterol: current use of cholesterol lowering medication in Million Women Study and UK Biobank. Variables used for depression: self-reported history of severe depression in the Million Women Study; derived mental state fields for major depression in UK Biobank.

eFigure 2. Study-specific Risk Ratios and 95% CIs for coronary heart disease and stroke first events in relation to living alone and to having little contact with family/friends/groups, subdivided by whether the first event was a hospital admission or a death without an associated hospital admission

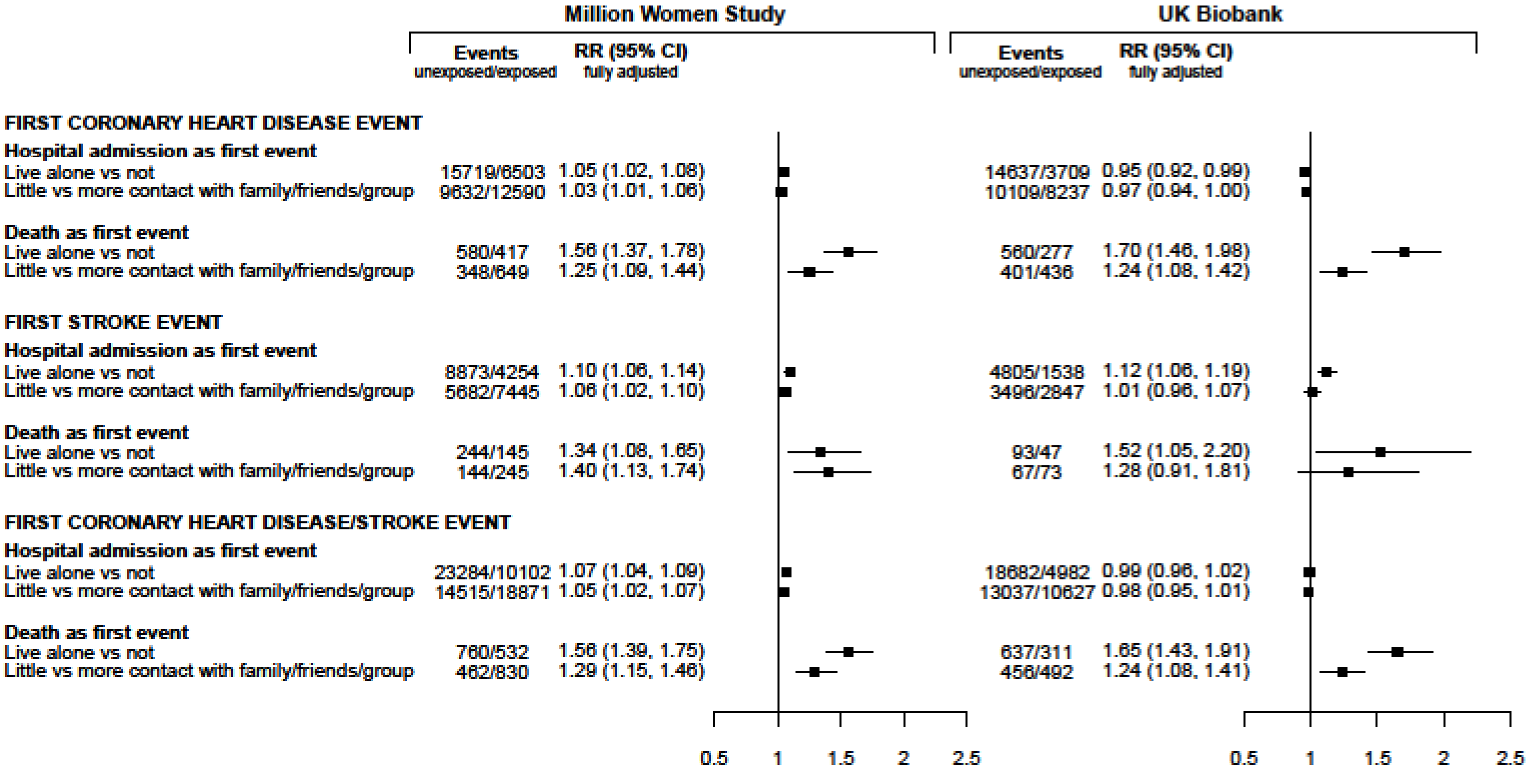

Fully adjusted for age, sex (in UK Biobank), region, deprivation, smoking, alcohol intake, body mass index, physical activity and self-rated health

eTable 5. Risk Ratios and 95% CIs for first coronary heart disease and stroke events in relation to three levels of social isolation, subdivided by sex and by whether the first event was a hospital admission or a death without an associated hospital admission, in UK Biobank only

	Women			Men		
	Events	RR (95% CI)		Events	RR (95% CI)	
**FIRST CORONARY HEART DISEASE EVENT**						
**Hospital admission as first event**						
Least isolated	2890	1.00		5168	1.00	
Moderately isolated	2795	0.99	(0.94,1.04)	4584	0.94	(0.91,0.98)
Most isolated	1058	1.05	(0.98,1.13)	1851	0.88	(0.84,0.93)
**Death without an associated hospital admission as first event**						
Least isolated	53	1.00		219	1.00	
Moderately isolated	83	1.52	(1.07,2.14)	272	1.26	(1.05,1.51)
Most isolated	40	1.94	(1.28,2.93)	170	1.74	(1.41,2.15)
**FIRST STROKE EVENT**						
**Hospital admission as first event**						
Least isolated	1139	1.00		1514	1.00	
Moderately isolated	1213	1.08	(1.00,1.18)	1413	1.01	(0.93,1.08)
Most isolated	445	1.11	(0.99,1.24)	619	1.04	(0.94,1.14)
**Death without an associated hospital admission as first event**						
Least isolated	23	1.00		28	1.00	
Moderately isolated	27	1.13	(0.64,1.97)	20	0.71	(0.40,1.26)
Most isolated	25	2.67	(1.49,4.80)	17	1.28	(0.68,2.40)

Fully adjusted for age, region, deprivation, smoking, alcohol intake, body mass index, physical activity and self-rated health

Membership of the Million Women Study Advisory Committee

Emily Banks, Valerie Beral, Lucy Carpenter, Carol Dezateux, Sarah Floud, Jane Green, Julietta Patnick, Richard Peto, Gillian Reeves, Cathie Sudlow.

Million Women Study coordinating centre staff

Simon Abbott, Rupert Alison, Krys Baker, Angela Balkwill, Isobel Barnes, Valerie Beral, Judith Black, Roger Blanks, Anna Brown, Andrew Chadwick, Dave Ewart, Sarah Floud, Kezia Gaitskell, Toral Gathani, Laura Gerrard, Adrian Goodill, Jane Green, Jane Henderson, Carol Hermon, Darren Hogg, Isobel Lingard, Sau Wan Kan, Nicky Langston, Kirstin Pirie, Gillian Reeves, Keith Shaw, Emma Sherman, Helena Strange, Siân Sweetland, Ruth Travis, Lyndsey Trickett, Clare Wotton, Owen Yang, Heather Young.

NHS Breast Cancer Screening Programme collaborating centres

The following NHS Breast Screening Centres took part in the recruitment and breast screening follow-up for the Million Women Study: Avon, Aylesbury, Barnsley, Basingstoke, Bedfordshire and Hertfordshire, Cambridge and Huntingdon, Chelmsford and Colchester, Chester, Cornwall, Crewe, Cumbria, Doncaster, Dorset, East Berkshire, East Cheshire, East Devon, East of Scotland, East Suffolk, East Sussex, Gateshead, Gloucestershire, Great Yarmouth, Hereford and Worcester, Kent, Kings Lynn, Leicestershire, Liverpool, Manchester, Milton Keynes, Newcastle, North Birmingham, North East Scotland, North Lancashire, North Middlesex, North Nottingham, North of Scotland, North Tees, North Yorkshire, Nottingham, Oxford, Portsmouth, Rotherham, Sheffield, Shropshire, Somerset, South Birmingham, South East Scotland, South East Staffordshire, South Derbyshire, South Essex, South Lancashire, South West Scotland, Surrey, Warrington Halton St Helens and Knowsley, Warwickshire Solihull and Coventry, West Berkshire, West Devon, West London, West Suffolk, West Sussex, Wiltshire, Winchester, Wirral, Wycombe.
